# The MANGO study: a prospective investigation of oxygen enhanced and blood-oxygen level dependent MRI as imaging biomarkers of hypoxia in glioblastoma

**DOI:** 10.3389/fonc.2023.1306164

**Published:** 2023-12-19

**Authors:** Caterina Brighi, David E. J. Waddington, Paul J. Keall, Jeremy Booth, Kieran O’Brien, Shona Silvester, Jonathon Parkinson, Marco Mueller, Jackie Yim, Dale L. Bailey, Michael Back, James Drummond

**Affiliations:** ^1^ Image X Institute, Sydney School of Health Sciences, The University of Sydney, Sydney, NSW, Australia; ^2^ Department of Radiation Oncology, Northern Sydney Cancer Centre, Royal North Shore Hospital, Sydney, NSW, Australia; ^3^ Institute of Medical Physics, School of Physics, The University of Sydney, Sydney, NSW, Australia; ^4^ Siemens Healthcare Pty Ltd, Brisbane, QLD, Australia; ^5^ Department of Neurosurgery, Royal North Shore Hospital, Sydney, NSW, Australia; ^6^ The Brain Cancer Group Sydney, St Leonards, NSW, Australia; ^7^ Centre for Health Economics Research and Evaluation, University of Technology Sydney, Sydney, NSW, Australia; ^8^ Department of Nuclear Medicine, Royal North Shore Hospital, Sydney, NSW, Australia; ^9^ Department of Neuroradiology, Royal North Shore Hospital, Sydney, NSW, Australia

**Keywords:** glioblastoma, hypoxia imaging, magnetic resonance imaging, radiotherapy, dose painting, treatment response assessment

## Abstract

**Background:**

Glioblastoma (GBM) is the most aggressive type of brain cancer, with a 5-year survival rate of ~5% and most tumours recurring locally within months of first-line treatment. Hypoxia is associated with worse clinical outcomes in GBM, as it leads to localized resistance to radiotherapy and subsequent tumour recurrence. Current standard of care treatment does not account for tumour hypoxia, due to the challenges of mapping tumour hypoxia in routine clinical practice. In this clinical study, we aim to investigate the role of oxygen enhanced (OE) and blood-oxygen level dependent (BOLD) MRI as non-invasive imaging biomarkers of hypoxia in GBM, and to evaluate their potential role in dose-painting radiotherapy planning and treatment response assessment.

**Methods:**

The primary endpoint is to evaluate the quantitative and spatial correlation between OE and BOLD MRI measurements and [^18^F]MISO values of uptake in the tumour. The secondary endpoints are to evaluate the repeatability of MRI biomarkers of hypoxia in a test-retest study, to estimate the potential clinical benefits of using MRI biomarkers of hypoxia to guide dose-painting radiotherapy, and to evaluate the ability of MRI biomarkers of hypoxia to assess treatment response. Twenty newly diagnosed GBM patients will be enrolled in this study. Patients will undergo standard of care treatment while receiving additional OE/BOLD MRI and [^18^F]MISO PET scans at several timepoints during treatment. The ability of OE/BOLD MRI to map hypoxic tumour regions will be evaluated by assessing spatial and quantitative correlations with areas of hypoxic tumour identified via [^18^F]MISO PET imaging.

**Discussion:**

MANGO (Magnetic resonance imaging of hypoxia for radiation treatment guidance in glioblastoma multiforme) is a diagnostic/prognostic study investigating the role of imaging biomarkers of hypoxia in GBM management. The study will generate a large amount of longitudinal multimodal MRI and PET imaging data that could be used to unveil dynamic changes in tumour physiology that currently limit treatment efficacy, thereby providing a means to develop more effective and personalised treatments.

## 1 Introduction

Glioblastoma (GBM) is the most aggressive type of brain cancer and carries an extremely poor prognosis, which is reflected in 5-year survival of 5% and an overall median survival time of approximately 17 months following first-line treatment (1). After standard of care treatment, involving maximal safe resection of the bulk tumour mass followed by adjuvant chemoradiation treatment, most tumours recur locally, highlighting the failure of current treatment approaches to sustain local control (2, 3). The large intra- and inter-tumour heterogeneity characterising GBMs and the ability of GBM cells to dynamically adapt during the course of treatment and develop treatment resistance mechanisms are causes of local treatment failure (4). As such, the future of improved treatment outcomes for GBM patients relies on personalised treatment strategies targeting known mechanisms of treatment resistance.

Hypoxia, which is often present in subregions of GBM tumours, is the leading cause of resistance to radiotherapy, is predictive of the local sites of recurrence and is associated with poor clinical outcomes in GBM (5–12). Current standard of care treatment does not account for tumour hypoxia. The ability to image tumour hypoxia at various stages of treatment offers opportunities to personalise and improve treatments for GBM patients (13). For instance, preoperative imaging of tumour hypoxia offers the opportunity for supramarginal resections in surgical planning beyond current neurosurgical standard of care guidelines (14). Identifying hypoxic regions by using specialised imaging potentially allows adaptive radiotherapy treatment planning and delivery strategies to selectively dose escalate to radioresistant tumour regions, thus increasing the likelihood of local control (10, 15). Identifying hypoxic tumour regions harbouring progression at follow up is key to intervening at an early stage of tumour recurrence and personalising therapy tailored to the tumour response to treatment (16).

Imaging of tumour hypoxia in routine clinical practice is challenging, as it requires [^18^F]MISO PET (13, 17), which is not available in most clinical centres (18). MRI, which is the core imaging modality in the management of GBM patients and is available at most clinical sites, offers a more cost effective and accessible means to integrating imaging biomarkers of hypoxia into clinical workflows (19, 20). Additionally, the combination of MRI screening with radiotherapy is becoming more common with the growth in integrated MRI-guided radiotherapy systems (21). In this clinical imaging study, we aim to validate oxygen enhanced (OE) and blood-oxygen level dependent (BOLD) MRI as imaging biomarkers of hypoxia at different stages of GBM treatment, and evaluate potential patients benefits arising from their integration into radiotherapy treatment planning and in treatment response assessment.

Several preclinical studies have already shown the ability of OE/BOLD MRI to identify areas of hypoxic tumour and use this information to improve cancer treatment and response assessment. For instance, Zhou et al. demonstrated that OE/BOLD MRI measurements correlate with levels of [^18^F]MISO uptake in hypoxic tumour of a rat prostate cancer model (22), thus showing its potential as an alternative, non-invasive imaging biomarker of hypoxia. White et al. have shown the ability of OE MRI to predict tumour response to radiation treatment in a preclinical model of prostate cancer, thus showing the potential of OE MRI for adaptive radiotherapy treatment strategies (23). This finding was further confirmed by Arai et al., who demonstrated the predictive value of OE MRI in response to radiation treatment in a rat model of prostate cancer (24). The authors additionally showed an improvement in tumour control resulting from the delivery of a 10 Gy boost of radiation dose to hypoxic tumours identified with OE MRI (24). Beeman et al. have demonstrated that OE MRI has the ability to differentiate progressing tumour lesions from radiation necrosis lesions in a preclinical glioma study (25), showing OE MRI potential for improved response assessment applications. Finally, in a recent clinical study Lickliter et al. demonstrated the utility of OE MRI to assess tumour response to the administration of a novel hypoxia modifying agent in combination with standard chemoradiation therapy in GBM patients (26).

Overall, by performing this study, we expect to provide a non-invasive, more effective means to adapt treatment to the individual tumour characteristics of each patient, that can be accessible to many clinical centres worldwide.

## 2 Methods and analysis

### 2.1 Study objectives

**Figure 1:**
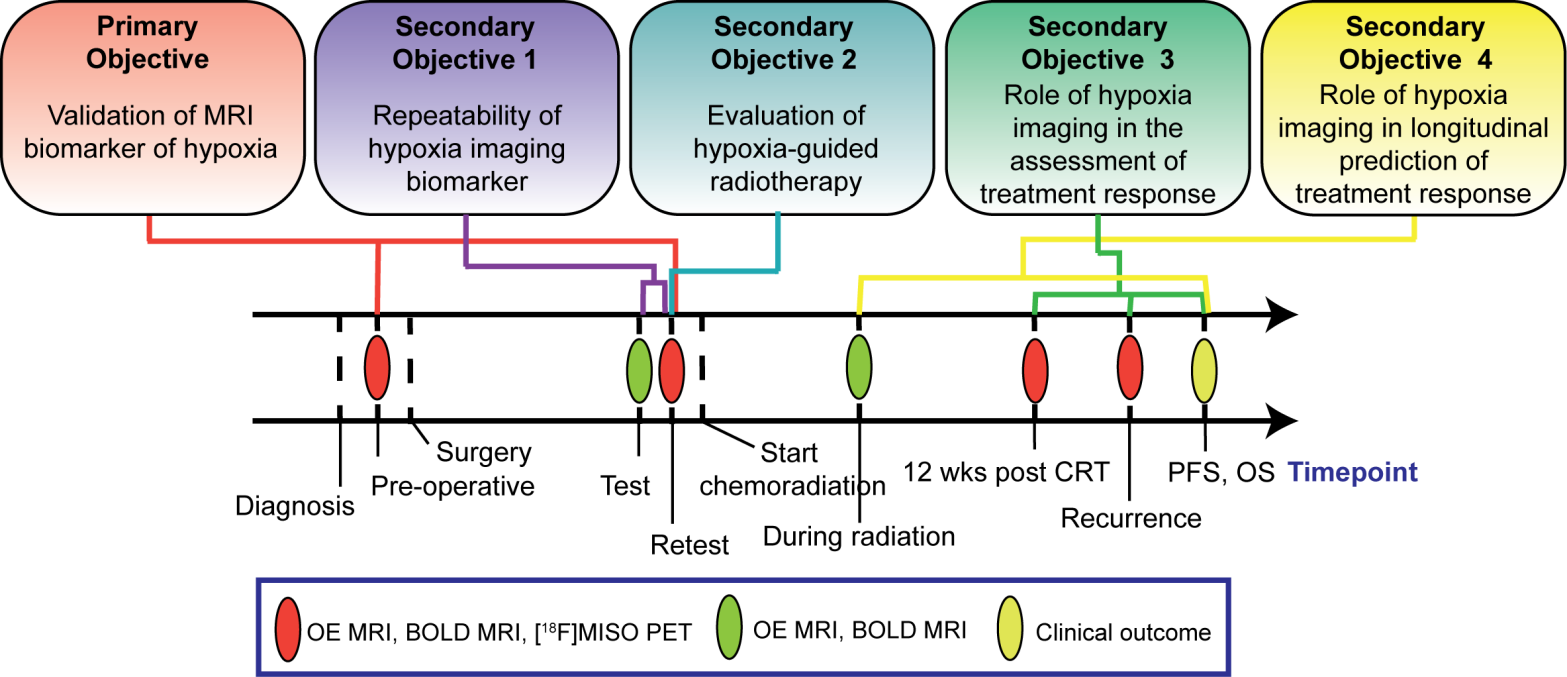
Diagram of study objectives and study interventions timeline.

The study has one primary and four secondary objectives (**Figure 1**). The primary objective is to evaluate the spatial and quantitative correlations between OE and BOLD MRI measurements of hypoxia and [^18^F]MISO values of uptake in the tumour once before patients receive surgery and once prior to commencement of chemoradiation treatment. The secondary objectives are: 1) to evaluate the repeatability of the MRI biomarkers of hypoxia by means of test-retest scans acquired prior to patients commencement of chemoradiation treatment; 2) to develop a framework to integrate image-derived tumour hypoxia information into radiation treatment plans and estimate the potential clinical benefits of this approach compared to conventional radiation treatment; 3) to evaluate the ability of MRI biomarkers of hypoxia to assess tumour response to chemoradiation treatment; 4) to evaluate whether changes in the MRI biomarker of hypoxia observed during chemoradiation treatment are predictive of patient overall survival. Secondary objectives 3 and 4 of this study are exploratory.

### 2.2 Study hypotheses

***Primary objective:*** MRI measurements of tumour hypoxia levels directly correlate to measurements of [^18^F]MISO PET uptake.

***Secondary objective 1:*** MRI measurements of hypoxia in the tumour volume acquired in the test-retest scans have excellent repeatability.

***Secondary objective 2***: Dose-painting radiotherapy plans that target areas of hypoxic tumour with high doses of radiation will result in improved modelled local tumour control probability and minimal alteration to normal tissue dose tolerances compared to standard radiotherapy plans.

***Secondary objective 3***: MRI measurements of tumour hypoxia at 12 weeks post chemoradiation treatment completion spatially overlap with sites of local recurrence and negatively correlate with patient overall survival time.

***Secondary objective 4***: Increases in measurements of tumour hypoxia observed during chemoradiation treatment negatively correlate with patient overall survival time.

### 2.3 Study outcome measures

#### 2.3.1 Primary objective

Spatial correlation between hypoxic tumour volume (HTV) determined with MRI and [^18^F]MISO will be evaluated via measurements of Dice similarity coefficient. Dice similarity coefficients > 0.75 will be considered a good spatial correlation. Quantitative correlation of voxel-wise and HTV-average levels of hypoxia will be evaluated via measurement of the Pearson’s correlation coefficient. Correlation coefficients > 0.7 will be considered a strong correlation (27).

#### 2.3.2 Secondary objective 1

Repeatability of voxel-wise and HTV-average levels of hypoxia in the tumour, calculated as explained below for each hypoxia imaging biomarker, will be assessed via measurements of intraclass correlation coefficient (ICC). Repeatability is considered excellent, good, moderate or poor for ICC values > 0.9, 0.75-0.9, 0.5- 0.75 and< 0.5, respectively. Additionally, similarity between the HTV defined with the MRI biomarker at the two timepoints will be assessed via calculation of Dice similarity coefficient. Dice similarity coefficients > 0.9 will be considered a strong correlation.

#### 2.3.3 Secondary objective 2

The predicted patient benefits of the hypoxia-guided dose-painting radiotherapy plan will be compared with the actual patient outcomes following conventional treatment, by using metrics including modelled tumour control probability (TCP) and toxicity measurement to organs at risks and healthy brain (including equivalent uniform dose). Voxel-wise dose-painting prescriptions within the conventional radiotherapy targets will be escalated from 60 Gy based on the level of hypoxia determined by the selected MRI imaging biomarker according to the method previously described by Toma-Dasu et al. (28). Targets delineation and constraints to organs at risk as per ESTRO-EANO guidelines (29) will be set equally for the optimisation of the conventional and dose-painting plans. Success for this objective will be achieved if the dose-painting plans result in improved TCP by more than 15% over conventional treatment, while dose metrics to organs at risk remain similar.

#### 2.3.4 Secondary objective 3

Spatial correlation between HTV after completion of chemoradiation treatment and volume of recurrent tumour at relapse evaluated with standard anatomical imaging will be evaluated via measurements of Dice similarity coefficient. Dice similarity coefficients > 0.9 will be considered a strong spatial correlation. Correlation between the percentage of HTV after completion of chemoradiation treatment and clinical outcome will be evaluated by means of hazard ratio obtained from Cox regression. A hazard ratio > 1 (p<0.05) will indicate that the HTV at suspected recurrence is associated with worse patient overall survival.

#### 2.3.5 Secondary objective 4

Correlation between the percentage change of HTV during treatment and patient clinical outcome will be evaluated by means of hazard ratio obtained from Cox regression. A hazard ratio > 1 (p<0.05) will indicate that the increase in HTV during treatment is associated with worse patient overall survival. Given the known prognostic role of sex and age on GBM overall survival (30–32), sub-analyses assessing whether HTV increase during treatment has a sex/age-specific impact on overall survival will be performed.

### 2.4 Study design

This study is a non-randomised, uncontrolled, non-interventional, prospective imaging study.

**Figure 2:**
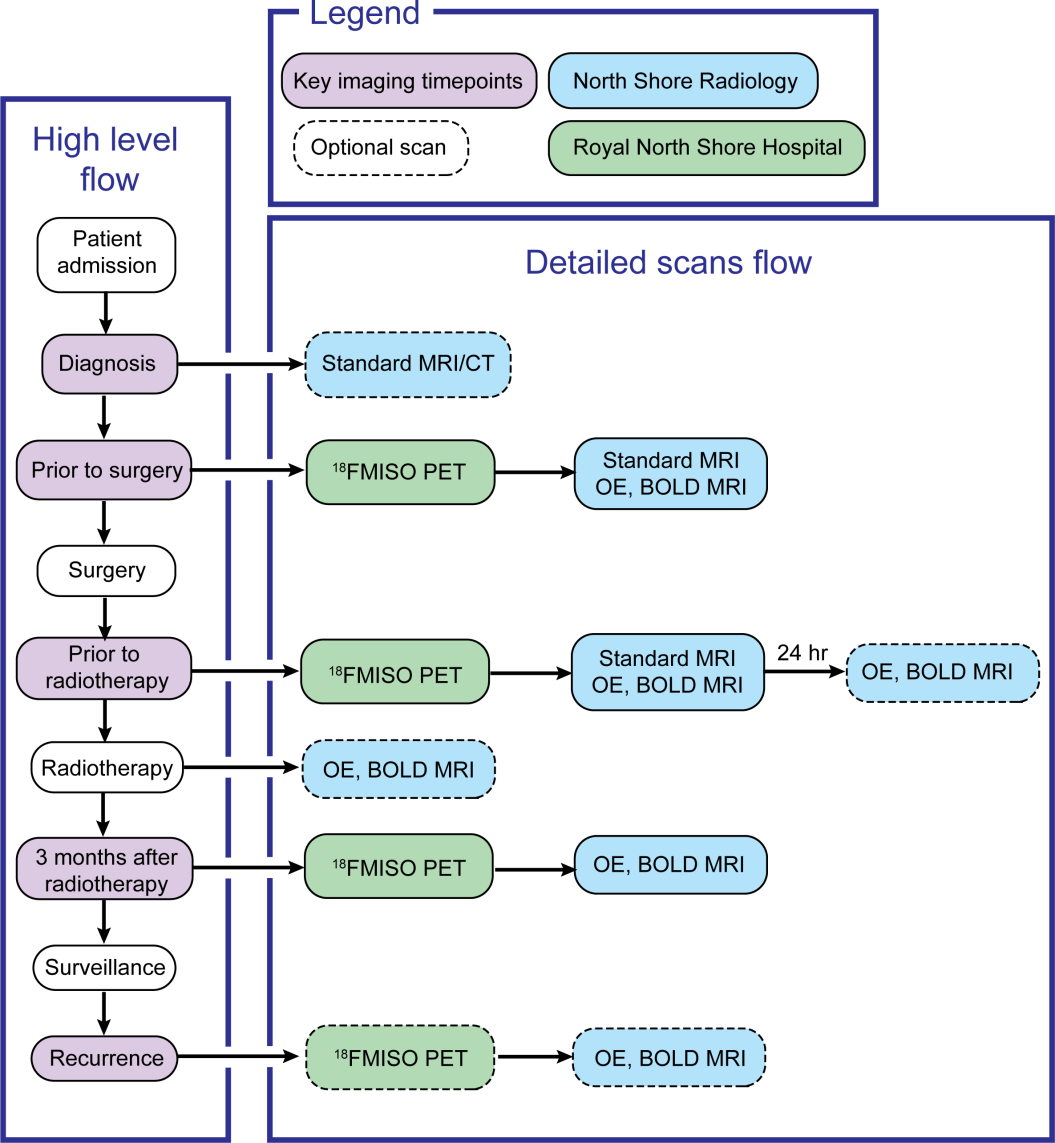
Diagram of study interventions flowchart.

This study will enrol patients with radiological diagnosis of grade IV glioma and for whom a diagnosis of glioblastoma, IDH wild type is confirmed on neuropathological exam, according to the WHO 2021 glioma classification criteria (33). Patients fulfilling the inclusion criteria will receive standard of care treatment according to the Stupp protocol (34), with the addition of OE and BOLD MRI and [^18^F]MISO PET scans to routine diagnostic protocols (**Figure 2**). MRI scans employing OE and BOLD sequences, as well as [^18^F]MISO PET/CT scans, will be acquired for patients pre-operatively at diagnosis, prior to commencement of chemoradiation treatment (3-4 weeks post-operative) and 12 weeks following completion of radiotherapy. Supplementary OE and BOLD MRI scans will also be performed in a test/retest setting prior to commencement of radiotherapy as well as during radiotherapy if the patients are amenable. If there is suspected recurrence on long term MRI surveillance, patients will be invited for repeat OE/BOLD MRI and [^18^F]MISO PET/CT scans. [^18^F]MISO PET imaging will be used to establish ground truth presence of hypoxia in the tumour. Existing correlations between MRI-derived levels of hypoxia and hypoxia levels assessed via [^18^F]MISO PET uptake will be evaluated (35–39). This study will require recruitment of a sample of 20 patients, as in previous clinical trials (NCT03716986, NCT02466828 and NCT00906893).

### 2.5 Study setting

In total, two sites will be utilised on the Northern Sydney Local Health District, Sydney, NSW, Australia: Royal North Shore Hospital (RNSH) and North Shore Radiology (NSR). Patients presenting with radiological symptoms of high-grade glioma at either of these sites will be presented with the opportunity to enrol in this study. This study is expected to recruit 20 patients. Following initial recruitment, NSR will be the site at which all the MRI scans will be performed, while RNSH will be the site at which the PET scans will be performed. Consent procedures may be performed at either site.

### 2.6 Selection of subjects

#### 2.6.1 Inclusion criteria

Male and female patients aged 18 years or older with suspected high-grade glioma at initial radiological examination and confirmed neuropathological diagnosis of glioblastoma, IDH wild type according to the WHO 2021 glioma classification criteria (33) will be recruited. Qualifying patients must have an Eastern Cooperative Oncology Group (ECOG) performance status score of 0-2, be available for scanning on two separate days and provide written informed consent for participation in this trial once eligibility is met.

#### 2.6.2 Exclusion criteria

- Women lactating, pregnant or of childbearing potential who are not willing to avoid pregnancy during the study
- Patients with a history of severe renal disease(s) (eGFR<20) who cannot tolerate gadolinium chelate contrast agents
- Neuropathological diagnosis not consistent with glioblastoma IDH wild type
- Geographically remote patients unable to agree to imaging schedule
- Patients who have received anti-VEGF monoclonal antibody therapy within 3 months prior to recruitment
- Patients with a history of psychological illness or condition such as to interfere with the patient’s ability to understand the requirements of the study
- Patients with significant cardiac or pulmonary disease including cardiac arrythmias or Chronic Obstructive Pulmonary Disease (COPD) that are unable to tolerate high flow O_2_ for oxygen contrast.
- Patients taking carbonic anhydrase inhibitors (Acetzaolamide)
- History of glaucoma
- Any contraindication to MRI imaging.

### 2.7 Interventional methods

#### 2.7.1 MRI protocol

**Figure 3:**
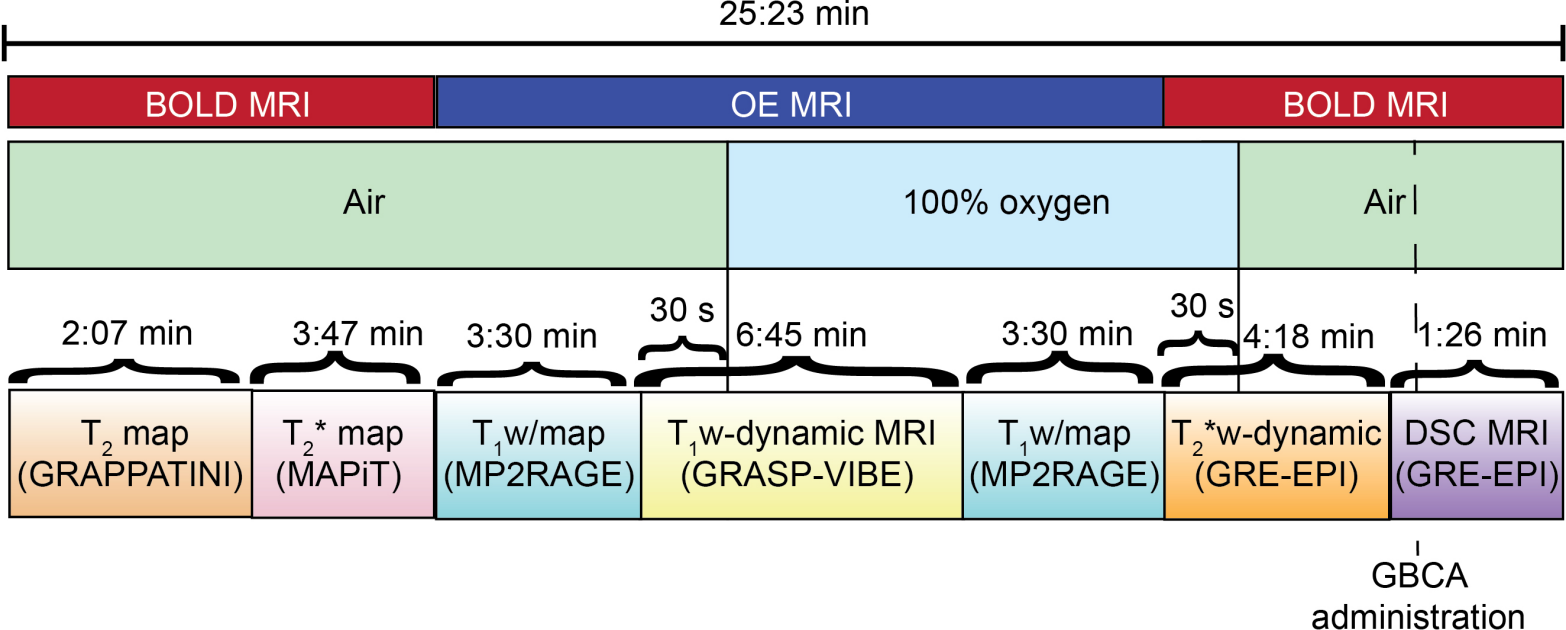
Schematic of oxygen delivery protocol and MRI sequences acquisition.

At each of the imaging timepoint determined in the study design patients will undergo the following MRI scans (**Figure 3**):

- OE MRI: including T_1_ mapping sequence prior to oxygen challenge, T_1_-weighted dynamic sequence (40) during oxygen challenge, T_1_ mapping sequence during oxygen challenge;
- BOLD MRI: T_2_ mapping sequence, T_2_* mapping sequence, T_2_* dynamic sequence during oxygen switch off, DSC sequence with single bolus of gadolinium-based contrast agent.

MRI scans will be acquired on Siemens 3T systems (Vida) with a 64-channels head coil.

Details of the MRI sequences acquisition parameters are collected in **Table 1**. T_1_-weighted dynamic imaging will be performed with a golden-angle radial sparse parallel-volumetric interpolated breath-hold (GRASP-VIBE) that permits reconstruction of image volumes at retrospectively determined temporal resolution (40). The DSC MRI sequence will be acquired to characterize tissue perfusion, which will be important in the analysis of MRI/PET images to identify regions of the tumour where hypoxia is not induced by poor tissue perfusion (41).

**Table 1:** MRI scan protocol acquisition parameters on a Siemens 3T scanner.

Parameter	T_1_ mapping + T13D (MP2RAGE)	T_1_w-dynamic (GRASP-VIBE)	T_2_ mapping (GRAPPATINI)	T_2_* mapping (MAPiT)	T_2_*w- dynamic (GRE-EPI)	DSC (GRE-EPI)
**Acquisition time**	3:30 min	6:45 min	2:07 min	3:47 min	4:18 min	1:26 min
**Resolution (mm^3^)**	1×1×1	1×1×1	0.7×0.7×4.0	1.7×1.7×4.0	1.7×1.7×4.0	1.7×1.7×4.0
**Matrix size**	240×256x192	256×256x104	320×240x30	128x128x30	128x128x20	128x128x20
**TR (ms)**	5000	4.51	5000	880	1230	1230
**TE (ms)**	2.9	1.65	16 TEs, TE1 = 10 ms, TE step=10 ms	12 TEs, TE1 = 6 ms, TE step=5 ms	30	30
**Flip angle**	0°	2°	180°	30°	90°	90°
**Number of averages**	1	1	1	1	1	1

During OE MRI acquisition patients will sequentially breathe medical air and 100% O_2_ (up to 15 l/min) through nasal prongs or oxygen mask. During DSC sequence acquisition, patients will be injected intravenously with a dose of 0.1 mmol/kg gadolinium-based contrast agent (GBCA) at a rate of 3-5 ml/s.

#### 2.7.2 MRI analysis

**Figure 4:**
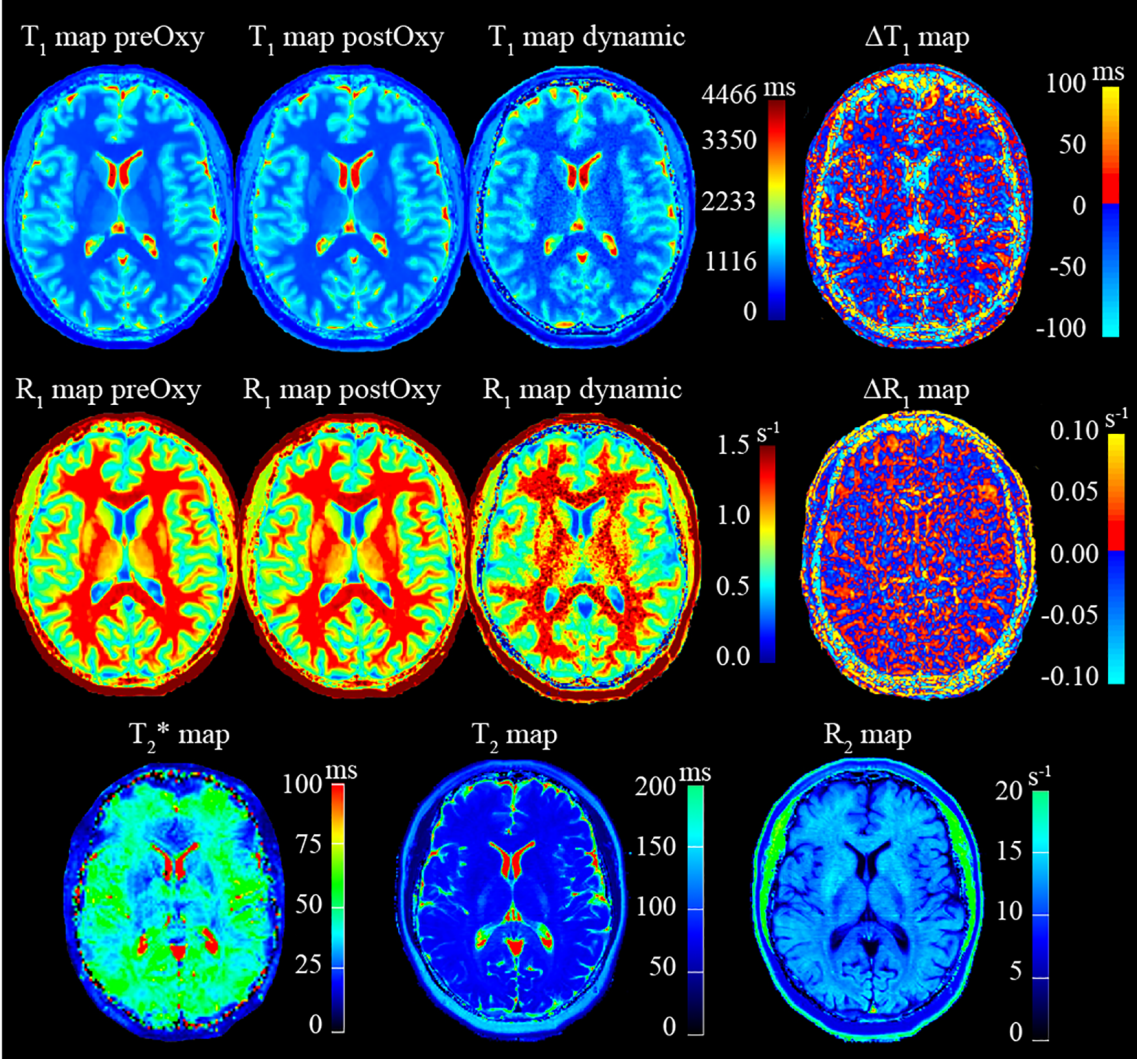
Example of quantitative images obtained from MRI protocol acquisition on a healthy volunteer.

Quantitative T_1_, T_2_ and T_2_^*^ maps will be derived from in-line processing of the T_1_-mapping, T_2_-mapping and T_2_^*^-mapping sequences, respectively, available on the scanner as part of the Siemens license agreement. Examples of quantitative images obtained from a healthy volunteer are shown in **Figure 4**. T_1_ maps for each timepoint of the T_1_-weighted dynamic acquisition will be calculated from static T_1_ maps by assuming that signal changes are due to T_1_ effects only, which is reasonable for sequences with very short echo times. These T_1_ maps calculations will be performed via an in-house software and a commercially available brain tumour imaging software, IB DCE (Imaging Biometrics LLC, Elm Grove, WI, USA). Cerebral blood flow (CBF) and cerebral blood volume (CBV) maps will be derived from kinetics modelling of the DSC data performed using the industry-leading brain tumour imaging software, IB Neuro (Imaging Biometrics LLC, Elm Grove, WI, USA).

A region of interest (ROI) will be delineated with a semi-automated tool and checked by an experienced neuroradiologist on the T_2_-weighted image as the hyperintense regions around the tumour. The ROI will be propagated to the T_1_, T_2_, T_2_^*^ and CBF maps. Voxel-wise and median values of R_1_, R_2_, R_2_^*^ and CBF within the ROI will be calculated from the quantitative T_1_, T_2_, T_2_^*^ and CBF maps, respectively. The reproducibility, repeatability and accuracy of the quantitative-MRI protocol will also be assessed using guidelines and a phantom developed by the National Institute of Standards and Technology and the International Society of Magnetic Resonance in Medicine (42).

On one hand, HTV will be defined via OE MRI as the proportion of voxels within the ROI being refractory in R_1_ for O_2_ challenge [i.e. ▵R_1_ ≤ 0, where ▵R_1_ = R_1_ (O_2_) – R_1_ (air)] and well-perfused (i.e. CBF > 0), as previously determined by experts in the field (36, 41, 43, 44). ▵R_1_ will be calculated in two ways. First, it will be calculated from the R_1_ maps obtained from the T_1_-mapping sequences acquired before [R_1_ (air)] and during O_2_ breathing [R_1_ (O_2_)]. Second, it will be calculated from T_1_ maps derived from the T_1_-weighted dynamic acquisition, such that R_1_ (air) is derived from the average of the first two R_1_ maps acquired at baseline while breathing air and R_1_ (O_2_) is derived from the last four R_1_ map acquired during oxygen breathing. ▵R_1_ values obtained with the two approaches will be compared. The approach which will provide the most reliable ▵R_1_ values in the test-retest scan across the entire patient cohort will then be selected to calculate the HTV. Voxel-wise ΔR_1_ values in the HTV will be converted into values of partial pressure of oxygen (pO_2_) using a similar approach to the one used by Chakhoyan et al. (35), which exploits known values of pO_2_ in normal appearing white and gray matter and uses these values to calibrate the pO_2_ values in the tumour. This approach will provide a way to quantitatively measure voxel-wise levels of hypoxia.

On the other hand, HTV will be defined via BOLD MRI by using a previously established method to estimate relative oxygen extraction fraction (rOEF) in human gliomas from T_2_, T_2_^*^ and CBV maps (37). Briefly, the transverse relaxation due to local magnetic field inhomogeneities in a blood vessel network, $R_{2}^{'}$ , will be calculated within the ROI from T_2_ and T_2_^*^ maps according to Equation 1:


(1) $$R_{2}^{'}=R_{2}^{*}-R_{2}=\left(\frac{1}{T_{2}^{*}}-\frac{1}{T_{2}}\right)$$

Then, the Yablonskiy and Haacke model will be applied to derive the oxygen extraction fraction (OEF) according to Equation 2:


(2) $$OEF=\frac{R_{2}^{'}}{\zeta \cdot \gamma \cdot \frac{4}{3}\cdot \pi \cdot \Delta \chi \cdot B_{0}}$$

where ζ is the venous fraction of the blood network and approximately equals the venous blood volume (~0.75.CBV), $B_{0}$ is the magnetic field strength, γ is the gyromagnetic ratio of value 2.675 . 10^8^ s^-1^ T^-1^, △χ is the susceptibility difference between deoxygenated and oxygenated blood $△\chi =△\chi_{0}\cdot Hct=0.924\cdot 10^{-7}$ , which is calculated from the susceptibility difference between fully deoxygenated and fully oxygenated hemoglobin $△\chi_{0}=0.264\cdot 10^{-6}$ and the small-vessel hematocrit (Hct = 0.35). As the absolute quantification of venous CBV in patients is not feasible, the venous blood vessel fraction ζ is approximated by CBV and the remaining factors are grouped into a constant $c=\gamma \cdot \frac{4}{3}\cdot \pi \cdot △\chi \cdot B_{0}=317\;s^{-1}$ . Using these simplifications, rOEF can be calculated according to Equation 3:


(3) $$rOEF=\frac{R_{2}^{'}}{CBV\cdot c}=\left(\frac{1}{T_{2}^{*}}-\frac{1}{T_{2}}\right)\cdot \frac{1}{CBV\cdot c}$$

To derive the HTV, a hypoxia rOEF threshold (rOEF_hypoxia_) will be defined from the mean, rOEF_mean_, and the standard deviation, rOEF_SD_, of the tissue within the ROI, such that rOEF_hypoxia_ = rOEF_mean_ + rOEF_SD_. Finally, the HTV will be defined by all voxels within the ROI for which rOEF> rOEF_hypoxia_. Hypoxia levels in voxels belonging to the HTV will be measured by rOEF values.

To facilitate the assessment of spatial and quantitative voxel-wise correlations in HTV and in levels of tumour hypoxia defined by the different imaging biomarkers, all MRI images will be resampled into the spatial resolution of the MRI image with the highest resolution by linear interpolation of voxel-wise intensity values.

#### 2.7.3 PET imaging protocol

At multiple timepoints determined in the study design (i.e. pre-op, once prior to commencement of chemoradiation and at suspected recurrence) the patients will also undergo a [^18^F]MISO PET/CT scan. Patients will be requested to come to the department 2 hours prior to the scan session. The amount of [^18^F]MISO given will be 370 MBq administered intravenously approximately 90 min prior to imaging. The PET data will be reconstructed to provide trans-axial images using standard protocols and the lesion uptake will be evaluated.

#### 2.7.4 PET imaging analysis

PET data will be reconstructed with CT data acquired simultaneously. To quantify the radioactivity concentrations in the PET images as standardised uptake values (SUV), raw PET images will be decay corrected to the point of tracer injection using a fluorine-18 half-life of 109.77 min. PET images will be co-registered to the MRI images acquired at the same timepoint of the study via rigid co-registration of the simultaneously acquired CT image to the T_2_ map. This way the ROI delineated on the T_2_ image can be applied to the PET SUV image and be used for quantification of lesion [^18^F]MISO uptake. The primary measures to quantify lesion [^18^F]MISO uptake will be maximum standardised uptake value (SUV_max_) and tumour-to-background ratios (TBR). TBR images will be derived by normalisation of the SUV image by the mean SUV value calculated in a region of interest delineated in the contralateral healthy brain. The HTV will be defined by all voxels within the tumour ROI with a TBR ≥ 1.2 - threshold value shown to optimally distinguish hypoxic tumour from normoxic brain tissue at 90 min post tracer injection in a previous GBM study (45) – and DSC MRI-derived CBF>0 – indicating well-perfused tissue. Hypoxia levels in voxels belonging to the HTV will be measured by values of pO_2_ derived from TBR values using the above mentioned approach used by Chakhoyan et al. (35).

Finally, to facilitate the assessment of spatial and quantitative voxel-wise correlations in HTV and in levels of tumour hypoxia defined by the different imaging biomarkers, [^18^F]MISO TBR images will be resampled into the spatial resolution of the MRI image with the highest resolution by linear interpolation of voxel-wise intensity values.

#### 2.7.5 Preparation and administration of [^18^F]MISO

Fluorine-18 will be sourced from a local PET radiopharmacy (Cyclotek NSW, Lucas Heights, Sydney). [^18^F]MISO will be synthesized on-site at RNSH using the ABX Products PE-SC-12, PE-TBA-001.750, PE-VVIAL-016 and the SCINTOMICS GRP^®^ Synthesizer. Cartridge purification will be carried out as part of the automated synthesis procedure programmed by Scintomics and ABX. ITLC and HPLC quality controls will be carried out post-synthesis and prior to release for patient administration. The pass mark for both HPLC and ITLC is >95%. The average QC results achieved for radiotracers produced at the RNSH facility are >98%. Following purification and characterisation procedures, a diagnostic dose of approximately 370 MBq ± 10% of [^18^F]MISO will be administered to patients via intravenous injection.

### 2.8 Data collection, storage, confidentiality and retention

There will be general information recorded about the patient including patient size (height and weight).

The following radiographic and radiotherapy-specific data will be collected for each patient:

- MRI images as DICOM series and raw k-space data in the proprietary vendor format
- [^18^F]MISO PET and CT data and reconstructed images as DICOM series
- Radiotherapy planning contours
- Radiotherapy treatment plans
- Clinical data including progression-free survival and overall survival

Patients enrolled in the study will receive a trial ID. The data saved for the trial will be under this de-identified trial ID. A separate key of the subject study number and their medical record number will be securely stored by the clinical trials staff to allow re-identification if necessary. This master list for re-identification will remain at RNSH. Only the clinical trials staff will have the ability to re-identify subjects. The data will be stored for 15 years as per Good Clinical Practice (GCP) guidelines. Imaging and clinical outcome data will be transferred from RNSH to the University of Sydney using a password protected cloud-based file share solutions from Citrix ShareFile, in accordance with the study site’s ethics and security allowances and protocols. The de-identified data will be stored for 15 years at the University of Sydney on a secure, password protected backed up storage drive (Research Data Storage). Only non-identifiable data may be made available for other scientific research, e.g., nonidentifiable data placed on a well-controlled university site, upon request. The data sharing platform is a secure on-line storage solution (“CloudStor”) provided through the University of Sydney. The data will be stored as a password-protected, encrypted file. In order to download or decompress the data, participating researchers will agree to the terms of use for the data, including that the data is not to be published or otherwise redistributed without the express consent of the original investigators.

### 2.9 Adverse event reporting and management

All adverse events related to the administration of oxygen for OE MRI and [^18^F]MISO for the PET imaging procedures will be reported to the Trial Management Committee and reviewed as to requirement for reporting to the institution ethics review board. Determination of the severity of adverse events will be consistent with Common Terminology Criteria for Adverse Events (version 5). The relationship between an adverse event and the investigational product will be determined by the Investigator. An event should be considered related to treatment if, in the clinical principal investigator’s medical judgment, there is a reasonable possibility that the event may have been caused by the study drug. The investigator will be responsible for ensuring that any adverse events observed by the investigator or reported by the subject are recorded in the subject’s medical record and reported.

Scans will be stopped if suspected adverse drug reactions, changes in vital signs, electrocardiogram, or clinical laboratory results are observed and these changes pose a significant health risk. Clinically or medically significant suspected adverse drug reactions, and serious adverse events considered to be related to study procedures will be followed until resolved or considered stable. Subjects withdrawn from the study will be treated as deemed appropriate by the treating physician. Safety follow-up procedures will be performed and the appropriate Clinical Research Facilities will be completed.

### 2.10 Statistical analysis

The sample size required for this study was calculated from a power analysis performed with the software GPower v.3.1. based on the hypothesis linked to the primary objective of the study. The hypothesis is that OE and BOLD MRI measurements of hypoxia within the tumour lesion will positively correlate (Pearson correlation coefficient > 0.7) to [^18^F]MISO values of uptake. The statistical test used in the power analysis was a correlation test using a point biserial model. Based on a Pearson correlation coefficient of 0.7, which was previously reported for the correlation of MRI measurements of hypoxia and the gold-standard immunohistochemical marker of hypoxia pimonidazole (27), the minimum sample size required to answer the hypothesis with 98% power and 5% two-tailed alpha, is 19. As such, up to 20 patients will be recruited for this study. A sample size of N=20 patients, which is large compared to the usual sample size used to estimate repeatability of ROI-averaged quantitative imaging measures (46), will allow us to test the hypothesis linked to secondary objective 1 - involving assessing the repeatability of OE/BOLD MRI measurements of hypoxia with the test-retest scan - with a specificity of at least 85% with 95% certainty (47); and the hypothesis linked to secondary objective 2 – involving the assessment of whether dose-painting radiotherapy plans result in improved TCP by more than 15% over conventional treatment - with 99% power and 5% one-tailed alpha (calculated with GPower v.3.1.).

## 3 Discussion

This diagnostic/prognostic imaging study will enable validation of the role of non-invasive MRI biomarkers of hypoxia that could be used to improve diagnosis, radiation treatment, prognosis and treatment response assessment of GBM patients. This study is ambitious in that it aims to characterise the evolution of tumour hypoxia levels during the whole course of patient management pipeline, with a plan to acquire MRI and PET/CT scans pre-operatively at diagnosis, prior to commencement of chemoradiation treatment, 12 weeks following completion of radiotherapy and at suspected diagnosis of recurrence, and supplementary MRI scans in a test-retest setting prior to commencement of radiotherapy and during radiotherapy. The supplementary MRI scans, which are required to test hypothesis for secondary objectives 1 and 4, will be considered optional based on patients’ status and compliance. Similarly, patients will be invited for repeat OE/BOLD MRI and [^18^F]MISO PET/CT scans only in case of suspected recurrence on long term MRI surveillance, meaning that there might only be a subset of patients whose data will be used to test hypothesis of secondary objective 3. Nonetheless, this study will generate a large amount of longitudinal multimodal MRI and PET imaging data that will be extremely valuable to unveil important dynamic changes in tumour physiology that currently limit treatment efficacy, thereby providing a means to develop more effective, personalised treatment strategies.

It is worth mentioning that one potential limitation in our study design is the challenge of including a selection criterium to ensure that the cohort of patients enrolled in the study exhibits different levels of hypoxia. This is important to ensure a more robust evaluation of the correlation between the OE/BOLD MRI biomarkers of hypoxia and [^18^F]MISO uptake levels. However, achieving such patients selection prior to enrolment in the study is neither trivial nor feasible in our study, because it would require quantification of hypoxia tumour levels via [^18^F]MISO PET scan, which is not performed as a routine standard-of-care diagnostic scan at our clinical centre. We mitigated the risk of this potential limitation by considering the typical incidence of hypoxia levels in GBM tumours. We looked at the results of a previously published clinical study quantifying regional levels of hypoxia in newly diagnosed GBM patients with [^18^F]MISO PET (12). This study enrolled 22 patients who had varying hypoxic tumour volumes (range: 0.1-129.3 cm^3^, median: 12.85 cm^3^) and heterogeneous levels of hypoxia (range T/B_max_: 1.2-4.2, median: 4.05). As we aim to recruit a similar number of patients with the same pathology, we assume to recruit a cohort of patients with similar hypoxia tumour volume/levels characteristics. It will certainly be important to report these metrics in our study results.

Moreover, we acknowledge that in our study we reserve the option of using either nasal prongs or a non-rebreather face mask for the delivery of oxygen gas in the OE MRI protocol. The choice for the oxygen delivery method will be linked to the feasibility of fitting the non-rebreather mask into the 64-channel MRI head coil that will be used in the study. The use of nasal prongs, which may be necessary for patients for whom a face mask would not fit comfortably into the head coil, could potentially limit the fractions of inspired oxygen (FiO_2_) to approximately 40%, hence resulting into a low OE MRI signal-to-noise ratio. We have mitigated this risk by testing our OE MRI protocol on a healthy volunteer who received oxygen delivery via nasal prongs. The ▵R_1_ measured in the nasal concha was 0.04 ± 0.04 s^-1^, which is comparable to the ▵R_1_ of 0.05 ± 0.02 s^-1^ observed in the same region of interest in healthy subjects in a similar OE MRI study using a non-rebreather oxygen mask (48). This result suggests that a similar hyper-oxygenation effect in the tissue could be achieved either by use of nasal prongs or non-rebreather face mask. Notably, validating that OE MRI can identify regions of tumour hypoxia by using nasal prongs for oxygen delivery would have higher impact in the widespread clinical translation of this method, as nasal prongs are safe, not limited by coil design or head shape, routinely used and widely available to all clinical centres.

## Ethics statement

All patients enrolled in the study are to provide written informed consent in accordance with institutional guidelines. Ethics approval for this study was obtained from the Northern Sydney Local Health District Human Research Ethics Committee on 15 April 2022: registration number 2021/ETH11794. This covers all participating clinical sites, including the Royal North Shore Hospital (RNSH) and North Shore Radiology (NSR).

## Author contributions

CB: Conceptualization, Data curation, Formal analysis, Investigation, Methodology, Project administration, Resources, Software, Supervision, Validation, Writing – original draft, Writing – review & editing. DW: Conceptualization, Funding acquisition, Investigation, Methodology, Resources, Software, Writing – review & editing. PK: Conceptualization, Writing – review & editing. JB: Conceptualization, Writing – review & editing. KO’B: Conceptualization, Resources, Software, Writing – review & editing. SS: Writing – review & editing, Project administration. JP: Funding acquisition, Writing – review & editing, Conceptualization. MM: Methodology, Writing – review & editing, Resources. JY: Funding acquisition, Writing – review & editing, Project administration. DB: Conceptualization, Methodology, Writing – review & editing. MB: Conceptualization, Funding acquisition, Writing – review & editing. JD: Conceptualization, Funding acquisition, Investigation, Methodology, Project administration, Resources, Writing – review & editing.
